# Community services' involvement in the discharge of older adults from hospital into the community

**DOI:** 10.5334/ijic.917

**Published:** 2013-09-18

**Authors:** Michelle Guerin, Karen Grimmer, Saravana Kumar

**Affiliations:** Division of Health Sciences, University of South Australia, Adelaide, Australia; Director International Centre for Allied Health Evidence, Division of Health Sciences, University of South Australia, Adelaide, Australia; International Centre for Allied Health Evidence, Division of Health Sciences, University of South Australia, Adelaide, Australia

**Keywords:** discharge process, hospital discharge, community services post-discharge support, older adults

## Abstract

**Background:**

Community services are playing an increasing role in supporting older adults who are discharged from hospital with ongoing non-acute care needs. However, there is a paucity of information regarding how community services are involved in the discharge process of older individuals from hospital into the community.

**Methods:**

Twenty-nine databases were searched from 1980 to 2012 (inclusive) for relevant primary published research, of any study design, as well as relevant unpublished work (e.g. clinical guidelines) which investigated community services' involvement in the discharge of older individuals from hospital into the community. Data analysis and quality appraisal (using McMaster critical appraisal tools) were undertaken predominately by the lead author. Data was synthesised qualitatively.

**Results:**

Twelve papers were eligible for inclusion (five randomised controlled trials, four before and after studies and three controlled trials), involving a total of 8440 older adults (>65 years). These papers reported on a range of interventions. During data synthesis, descriptors were assigned to four emergent discharge methods: Virtual Interface Model, In-reach Interface Model, Out-reach Interface Model and Independent Interface Model. In each model, the findings were mixed in terms of health care and patient and carer outcomes.

**Conclusions:**

It is plausible that each model identified in this systematic review has a role to play in successfully discharging different cohorts of older adults from hospital. Further research is required to identify appropriate population groups for various discharge models and to select suitable outcome measures to determine the effectiveness of these models, considering all stakeholders' involved.

## Introduction

In Australia, as in most Western countries, the mean age of the population is increasing, due to decreasing birth rates and increasing life expectancy. In Australia, the population of adults over the age of 65 years is estimated to reach 8.1 million by 2050, compared with only 3 million in 2010 [1, p. 5]. Despite many older adults living healthy active lives, ageing is associated with increased risk of disease, disability and complex care needs (e.g. chronic health conditions, limited social support and financial hardship) [1,2]. Consequently, older adults are high users of hospital care. In Australia, older adults aged 65 years and over accounted for 13.5% of the population in 2010, yet utilised 50% of acute hospital bed days. This is expected to increase to around 70% by 2050 [1,3].

To assist in managing the current and anticipated increase in demand on acute hospitals by older adults, it is imperative that older adults are assisted to leave hospital and return to the community in a safe, timely and effective manner [2,4]. To facilitate this process in Australia, hospital discharge polices and guidelines have been implemented variably across the nation [5–8]. Most policies appear to focus on the development of individualised discharge plans, which aim to reduce acute hospital costs by minimising the length of hospital stay and shifting non-acute care into the community [5–10]. For hospital-developed discharge processes to be effective, community services need to be engaged to provide important non-acute post-hospital care. Consequently, community services are playing an important and growing role in meeting older adults’ care needs throughout the discharge process and into the community [2,10].

Despite the increasingly important role of community services in the discharge process, research spanning the last 30 years has consistently highlighted key problems associated with collaboration between hospitals and community services to effectively discharge older adults [11,12]. These problems include poor communication between hospital staff [13,14], community service providers [15–17] and older adults [13,16,18]; delayed and inadequate assessments of discharge needs [13,19]; poor organisation of community services [16,17] and delayed community services involvement after discharge from hospital [19]. These problems are associated with increased hospital costs; lengthy hospital stays [19–21], increased rates of unplanned hospital readmissions [22] and compromised patient safety and satisfaction [15,17,20].

While there is a considerable volume of research detailing the problems associated with community service and hospital collaborations, there is a paucity of research which investigates the ways community services and hospital can work together, across the hospital–community interface, to successfully discharge older adults. To our knowledge, a systematic review, which focuses solely on community services' involvement in the discharge process across the hospital–community interface for older adults, has not previously been published.

The primary aim of this systematic review, therefore, was to identify and critically appraise the relevant literature detailing methods of community services' involvement in the discharge of older adults across the hospital–community interface.

The secondary aim of this review was to identify the most effective methods of community service involvement in the discharge process of older adults.

## Methods

### Definitions

For the purpose of this review, hospitals were defined as acute care hospitals, which were ‘capable of providing high-technology inpatient care and catering to admissions with acute medical and surgical problems; nursing homes, rehabilitation and community hospitals not providing high technology care are not included’ [23, p. 7].

Community was broadly defined as living outside of a permanent institution (e.g. hospital, residential care facility and prison).

Interventions provided across the hospital–community interface were defined as those ‘interventions delivered in both the hospital and community setting to the same patient during the process of discharge from inpatient hospital care. They key issue was that the intervention was not delivered in one setting in isolation from the other’ [23, p. 7].

#### Community services

Community services that assist in the discharge process have been previously defined as those services based in the community which cover the following types of services [24, p. 3]:

1. Interventions to maintain or optimise functional capacity and independence.
2. Slow-stream rehabilitation.
3. A case management role which is situated in the community.
4. Planned supported transfer of individuals from hospital to community.

### Criteria for considering studies for this review

#### Study design

This review included all relevant primary published research of any study design as well as relevant unpublished work, such as clinical guidelines.

#### Publication

Studies published in English between 1980 and July 2012 inclusive.

#### Participants

The studies had to report on adults aged 65 years and over, who had been admitted to an acute care hospital from the community and were returning to the community to live.

#### Geography

Studies from all countries were included.

#### Community services involvement

Each study had to describe the involvement of community services in the discharge process which spanned the hospital–community interface and key components of services and processes. These could include the following:

- Service design features (range of services, health professional(s) involved, location, relationships with other health service providers).
- Degree of integration and coordination across the hospital–community interface and with other service providers (including communication mode and referral pathways).
- Implementation and provision of post-discharge support.

#### Outcomes

Any outcome was reported.

### Study exclusion criteria

The literature excluded from review comprised the following:

- Letters to the editor, comments, editorials, abstracts only, books and book chapters, conference proceedings.
- Studies which investigated community services post-discharge.
- Studies with inadequate description of community services involvement (e.g. studies which simply stated community services were engaged) and that provided no detail of the involvement of the service across the hospital–community interface.
- Studies which included services that were delivered in the community but were not community-based (i.e. hospital outreach services that did not interface with community-based services).
- Studies addressing disease-specific adults, where the intervention was only applicable to that specific group.

### Search methods for identification of studies

This search aimed to capture a comprehensive list of published literature, and unpublished work reported in well-regarded repositories (international guideline sites, for instance), detailing the ways community services were involved in the discharge for older adults across the hospital–community interface. The search process included, keyword searches of electronic databases, hand searching of key journals, pearling of relevant references lists and citation searching of key papers.

Twenty-nine databases covering health and social sciences literature, unpublished (grey literature) and current publications were searched: Academic search elite; AARP Ageline; AMED; APAIS-Health; ATSI health; AustportMed; CINAHL; Current Contents Connect; EMBASE; Health Business Full text Elite; Health sources: Nursing/Academic Edition; Health and Society database; Humanities and Social Sciences Collections; Health Sciences: A SAGE Full-Text Collection; Index to Theses; MEDLINE; Meditext; Pre-cinahl; PsycInfo; Social Sciences Citation Index; Scopus; Cochrane Library; Scottish Intercollegiate Guidelines Network (SIGN); National Guideline Clearinghouse (NGC); UK Department of Health publications; National Institute for Health and Clinical Excellence Guidelines; Digital Dissertation; Metacrawler; Google Scholar.

Key journals: Age and Ageing, BMJ, Journal of the American Geriatrics Society, Medical Care Research and Review, Journal of Advanced Nursing and Journal of Ageing and Health were hand searched for relevant articles.

The reference lists of all included studies were searched for additional relevant literature.

Key papers identified in the search were read to identify any relevant literature. Key papers were those papers which discussed the topic of interest, but that did not meet the inclusion criteria of this systematic review (detailed above).

### Search terms

Search terms were devised to retrieve studies that included the concepts of ‘discharge planning’, ‘older adult’ and ‘community services’. Refer to Tables 1 and 2 for the search terms used.

These search terms were developed in three phases. The first phase collated the search terms reported in recent systematic reviews on discharge across the hospital–community [25–27]. The second phase was independent validation by the Senior University of South Australia Health Liaison Librarian on whether the proposed terms were comprehensive and complete. The third phase involved searching Medline database and scanning keywords used in similar studies.

*These search terms were added to the updated search (2011–2012) reflecting the broadening of terms utilised in the literature to discuss discharge planning.

An abridged search strategy was devised for databases where it was not possible to use the full-search strategy.

Truncation symbols, relevant to specific databases, were utilised to maximise search results.

## Methods of the review

Studies were assessed for relevance utilising a two-staged process.

- Title and abstract reviewed

The primary researcher (M.G.) reviewed all the titles and abstracts of the identified studies to determine their relevance. If it was unclear from the title and the abstract, if the study met the inclusion criteria, the full-text article was retrieved.

- Full-text article reviewed

The primary researcher (M.G.) and another reviewer independently read the introduction and methods section of full-text articles to determine whether they met the inclusion criteria. If the inclusion of the study could not be ascertained at this point the results section was then read. Any disagreement was resolved through discussion.

Where information was not clear, or further information was needed to determine the studies eligibility criteria, the study authors were contacted via email for further information.

### Data extraction

Data from all included studies were extracted by the primary researcher onto a specifically designed template. The template was designed, based on the aims of the systematic review and the data required in meeting the aims.

### Critical appraisal stage

The design-generic McMaster qualitative and quantitative critical appraisal tools were chosen for critical appraisal in this review, as it was anticipated that the literature identified in the search strategy may reflect either of these research designs.

The McMaster critical appraisal tools were not originally scored; however, for the purpose of this review, a scoring system was devised to enable the quality of the articles to be compared. A score of 1 was attributed to ‘yes’, 0 for no’ or ‘not addressed’, and items determined as ‘not applicable’ were not considered in the overall possible score. The McMaster quantitative critical appraisal tool has 15 quality criteria, 14 of which are quantifiable, permitting a total score of 14. This appraisal tool has 20 quality items, 18 of which are accessible, allowing a score out of 18. To enable comparison between studies (to take account of different denominators), raw scores were converted to percentages.

All articles were critically appraised by the primary researcher (M.G.) and a random selection of four articles was independently appraised by a second person (K.G.) to ensure inter-rater reliability.

## Results

### Description of studies

#### Search results

**Figure 1.**
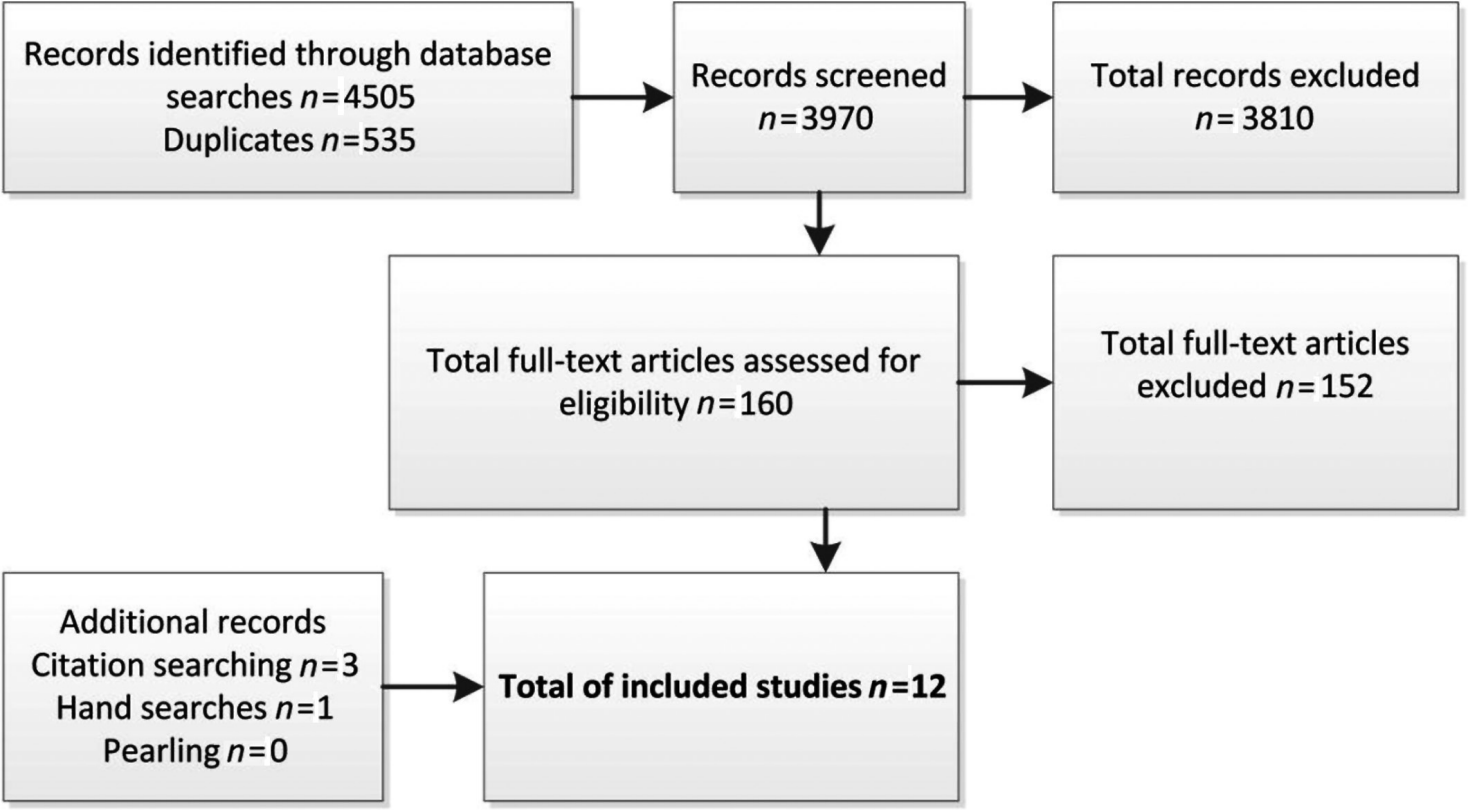
Flow chart of included and excluded studies.

The flow diagram of references included and excluded is presented in Figure 1, identifying that eight studies from the database searches fulfilled the inclusion criteria. The review of reference lists and hand searching yielded an additional four studies.

We identified 12 quantitative studies from four countries, involving a total of 8440 older adults (>65 years), most with complex chronic health conditions, frailty/deconditioning or identified at risk of readmission to hospital after discharge. Table 3 describes the studies.

#### Methodological quality of the studies

The methodological quality of the studies in this review varied, with critical appraisal scores ranging from 57 to 91%. There were no major disagreements in scoring between the reviewers.

Common methodological problems related to the sample (inadequate description of subjects (four studies), no sample size justification (five studies), lack of detail regarding validity and reliability of outcome measures utilised (five studies) and insufficient information regarding avoiding contamination and cointervention (six studies) (see Table 4).

There were additional methodological concerns identified by the primary researcher when reviewing the articles, which were not part of the formal critical appraisal process. In the majority of included studies, the intervention involving community services was compared with ‘usual care’; however ‘usual care’ was poorly described in majority of the studies. Common explanations of usual care included ‘after their discharge, the patients were allocated social and medical support according to prevailing criteria’ [28, p. 446], or ‘patients randomised to the control group received conventional medical care under the direction of their regular physician. Control group patients also received all standard hospital services, including dietary teaching and predischarged medication instructions’ [29, p. 271]. Poor description of usual care hindered the ability to identify the difference(s) between the intervention group and the usual care group and what potentially could be contributing to the difference(s) in outcome. Another methodological concern was that while the studies justified a need for improved discharge planning, all but three studies [30–32] did not justify why that particular intervention, over ‘usual care’, had been designed and researched. These methodological concerns impact on the applicability of the findings to inform further research and clinical practice.

## Models of community services' involvement in the discharge of older adults across the hospital–community interface

The included studies reported on a range of hospital and community services' involvement in the discharge process of older adults. Various interventions were discussed in these studies, including; nurses liaising with community services to arrange follow-up care [28,31,33]; specialised hospital teams coordinating older adults care across the interface [34]; hospital pharmacists organising care with community pharmacists [35]; community services assessing older adults in-hospital and arranging supporting following discharge from hospital [30,36] and hospital staff undertaking home visits after discharge while coordinating care with community service providers [32,37]. These interventions were generally compared to usual care, which, as previously noted, was generally poorly defined.

We distilled the interventions into four ‘general’ methods of community services' involvement in the discharge of older adults across the hospital–community interface:

- Virtual Interface Model
- In-reach Interface Model
- Out-reach Interface Model
- Independent Interface Model

### Virtual Interface Model (Denmark, Aus, Aus, UK, USA, USA)

**Figure 2.**
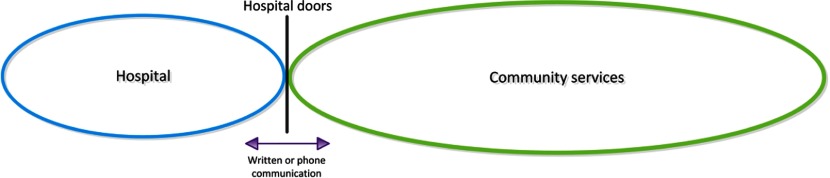
Diagram of Virtual Interface Model.

The predominant model of community services' involvement in the discharge process was the model by which hospital and community services staff remained in their respective environments (i.e. did not ‘physically’ cross the interface) and communicated across the interface through phone or written communication (fax or paper copy referrals). For the purpose of this review, this model was named the Virtual Interface Model (Figure 2). Six of the 12 studies operated within the Virtual Interface Model [28,29,34,35,38,39].

In this model, hospital staff were responsible for undertaking discharge assessments (either in the emergency departments or hospital wards), developing the discharge plans and referring to community services (via phone or written communication) at the point of discharge from hospital, or just prior. Hospital staff involved ranged from single hospital nurses [28] and hospital pharmacists [35], to multidisciplinary teams comprising of nurses, allied health professionals, social services and doctors [29,34,38,39].

Hospital staff referred to a variety of community services to support older adults post-discharge, ranging from local government (councils) [34,39] to community nursing [28,29,34,38,39]. In some instances, only one community service was engaged [29,35,38], while in other cases a range of community services was involved [28,34,39] to implement the discharge plans (refer to Table 5).

The interventions delivered by the community services after discharge from hospital varied in both number of visits and overall time. Certain community service interventions involved a one-off visit [28], while other interventions were more intensive, providing up to 90 days of support [29,38] (Table 5).

The outcomes of the Virtual Bridging Interface Model in relation to healthcare outcomes (hospital operational efficiencies and economic costs) and patient/carer were mixed, with inconsistent findings across the studies (Table 5).

### In-reach Interface Model (UK and Aus)

**Figure 3.**
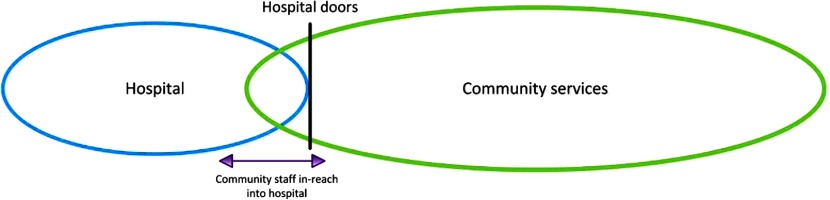
Diagram of In-reach Interface Model.

Two studies engaged community services in the discharge process by having community services situated in the acute care sector to undertake the discharge assessment(s) and develop the discharge plans [30,36] (Figure 3).

In this model, community services were responsible for assessing older adults, developing the discharge plans and transitioning them across the hospital–community interface. In the In-reach Interface Model, community services' were involved early in the discharge process and had greater input into discharge assessment and planning, compared with the other three models.

Cunliffe et al.'s [36] study employed a multi-disciplinary community team based in the hospital whose responsibility it was to assess the older adult, develop the discharge plans, transition the older adult across the interface and provide ongoing care in the community. Hegney et al.'s [30] Australian study had a single community nurse situated in the hospital who undertook the discharge assessments, developed the discharge plan and then referred directly to Home and Community Care Services (HACC) if eligible, and if not, to other suitable community providers (Table 6).

Information regarding how community services were initially engaged in the discharge process, when they were engaged and the length of intervention delivered in the community, was inconsistently reported in these two studies (Table 6). This impacts on the generalisability of these findings to inform further research and clinical practice.

Outcomes of the In-reach Interface model were inconsistent (Table 6). Both studies identified that the intervention was as safe as the usual care group, with no significant differences in mortality reported between the two groups.

### Out-reach Interface Model (USA, USA)

**Figure 4.**
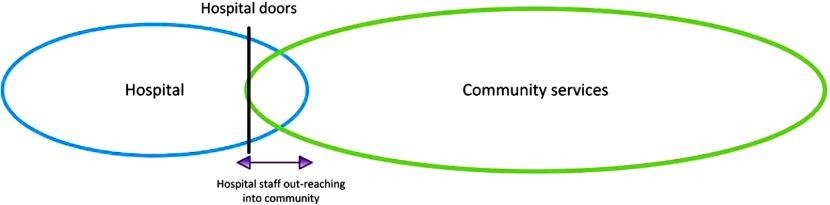
Diagram of Out-reach Interface Model.

Two of the studies in this review described a model of community service involvement whereby hospital staff crossed the hospital–community interface into the community, where they implemented certain aspects of the discharge plans [32,37] (Figure 4). During this implementation phase, hospital staff liaised with community services to deliver other aspects of the discharge plans (Table 7).

In this model, hospital staff (nurses or social workers) were responsible for assessing older adults’ discharge needs, developing discharge plans and implementing aspects of the discharge plan upon discharge from hospital.

Siu et al.'s [37] study employed a hospital nurse-led inter-disciplinary team whose responsibility it was to undertake discharge assessment and planning in hospital, with the nurse implementing aspects of the discharge plan in the community. Community services were engaged by the hospital nurses after they had completed their first home visit. The main community health professional to be engaged was the older adult's general practitioner. The general practitioner was sent a letter by the hospital detailing the older adult's interventions and gaining approval for other community service interventions, such as allied health (see Table 7). In Watkins et al.'s study [32], a social worker was employed to facilitate discharge assessment and planning in hospital and to support the patient in navigating the hospital post-discharge period. To assist with transitioning home, community services engaged prior to discharge included skilled nursing, allied health, housekeeping, transportation and medication reminders. Other typical services arranged by the social worker included equipment/aids, Meals on Wheels and community volunteer programs (Table 7).

In the Out-reach Interface Model, the main role of community services was to compliment the services provided by the hospital (e.g. nurse, social worker) and deliver services beyond the scope of this role (Table 7).

The outcomes of the Out-reach Interface Model were again mixed (Table 7). Sui et al. [37] demonstrated no significant impact on health care system and patient/client outcomes, with the exception of patient/client satisfaction, which was significantly less in the intervention group (Table 7). Watkins et al. [32], however, identified reductions in readmissions rates, increased quality of life scores and overall patient satisfaction.

### Independent Interface Model (USA)

**Figure 5.**
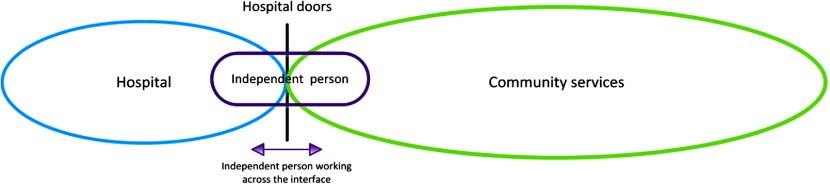
Diagram of Independent Interface Model.

Two studies included in this review involved an independent person (not employed by the hospital or community service) working across the hospital–community interface to facilitate the discharge of older adults from hospital into the community [31,33] (Figure 5).

In both studies exploring this model, the independent person employed to work across the interface was a nurse. The nurse's main roles included assessing the older adult in hospital, facilitating communication across the hospital–community interface and providing home visits once the person returned to the community. In this model, continuity of care with the nurse was maintained throughout the hospital stay and into the community.

Older adults facilitated to leave hospital and return home included those who were ongoing recipients of a home-based primary care program [31,33] and those with medical problems associated with high rates of hospitalisation [31]. Older adults were assisted to leave hospital and return home from emergency departments and hospital acute and long stay wards.

The key community service involved in providing care after discharge from hospital was the older adult's general practitioner. Other community services engaged to provide support in the community were allied health and home help services (Table 8). These services supported the older adult and complimented the ‘medical type’ services provided by the nurse. Community services in Ornstein et al.'s study [33] were engaged through computer notification. In Naylor et al.'s study [31] how and when community services were engaged was not reported (Table 8).

The outcomes of the Independent Interface Model were inconsistent. Ornstein et al. [33] demonstrated positive qualitative outcomes in terms of hospital staffs’ opinion of the programme. For example, the programme was considered to save hospital staffs’ time, streamline the discharge process and improve inpatient management. Yet in terms of health care efficiencies, and patient and carer outcomes, there were no significant differences. Naylor et al.'s study [31] demonstrated significant differences in relation to increased hospital efficiencies and reduced costs; however, there were no differences in patient outcomes (Table 8).

## Discussion

Despite the limited research into the ways community services can work with hospitals across the hospital–community interface, this review provides new information on the roles that community services are playing in the discharge of older adults from hospital. This review identified 12 studies which described four ‘general’ methods of community services' involvement in discharge, coined as follows:

- Virtual Interface Model
- In-reach Interface Model
- Out-reach Interface Model
- Independent Interface Model

### Virtual Interface Model

The Virtual Interface Model was the predominant model reported in this review, likely reflecting the traditional ‘hospital-centric’ approach to discharging older adults across the hospital–community interface [15,16]. In this model, hospitals assumed the lead role, with community services responsible for implementing the hospital's discharge plans.

### In-reach Interface Model

The In-reach Interface Model involved dedicated and appropriately funded community service staff working within the hospital. Their role involved arranging discharges and facilitating older adults’ transition across the hospital–community interface to their respective community service.

The In-reach Interface Model was utilised where the community service undertaking the assessment could usually provide all the needed ongoing care in the community. However, how this model would operate in practice, where a number of community services were required to meet the older adult's needs was not explored in either of the studies.

### Out-reach Interface Model

The Out-reach Interface Model was informed by two studies [32,37]. The focus of this model was on addressing older adults’ ongoing medical and social needs. Community services' role was to support the older adult and carer(s) in functional and health-related tasks, which were beyond the scope of the hospital staff.

### Independent Interface Model

Two studies identified in this review operated within this model [31,33]. In these studies, specifically funded independent nurses were employed to work across the hospital–community interface, providing services in both the hospital and community settings. The care delivered in this model was focused predominately on meeting older adults’ medical needs.

### Outcomes

Across the included studies, 14 outcomes using a range of outcome measures were reported. The most commonly reported outcomes focused on health care outcomes (e.g. readmission rates, mortality, hospital length of stay and service utilisation and costs) and patient and carer outcomes (e.g. patient and carer satisfaction, functional status and quality of life/well-being).

The effectiveness of the four models identified in this review could not be determined for any outcome measure. This was due to the limited number of studies identified under each model and our inability to pool the data due to the variability in the outcomes collected, the heterogeneous outcomes measures used and the time periods over which outcomes were measured.

The outcomes reported upon in this review appropriately attempted to measure the impacts of their interventions in relation to the commonly reported consequences of problematic discharge in terms of hospital and patient and carer outcomes. However, none of the studies attempted to measure the impact of their intervention on community services. In considering the consequences of problematic discharges on community services, reported in the literature [16,17,31,33], potentially important outcomes to consider could include the following: the number of referrals deemed appropriate where community services could meet the person's needs, the ability to mobilise services and equipment prior to the person's return home and the number and types of changes made to the discharge plans developed by the hospital staff and the implications on resources for community service. Measuring these types of outcomes would enable greater insight into community services' involvement in the discharge process and the resultant outcomes.

### Informing clinical practice and further research

In terms of clinical practice, it is possible that all four models of community service involvement in the discharge process, which were distilled from this review, may have a place in the discharge of older adults from hospital into the community. The key to further research is likely to be in identifying which models are best-suited to specific patient and carer groups (based on their discharge needs) and the supportive funding models. For example, in considering these models, it seems plausible that the Virtual Interface Model could be a suitable model for discharging older adults across the interface with relatively ‘simple’ discharges. That is, those older adults where minimal negotiation between hospital and community service providers is required and where community services have the capacity to act upon the referrals in a timely manner. The Outreach Interface Model, on the other hand, may be appropriate for older adults who are discharged home with specialised needs (e.g. medical) which can only be delivered by hospital staff. The role of community services in this model would be to provide support to the older adult, complementing the ongoing specialist care provided by the hospital staff. If we consider the In-reach and the Independent Interface Models, in terms of clinical practice, both these models may be likely to address the needs of those older adults with ‘complex’ discharge needs. Namely, older adults who require considerable negotiation of services across the interface, the engagement of a number of community services and/or the planning and implementation of services and supports which take time and can be complex to arrange (e.g. home modifications, and community care packages). The specific discharge model(s) used (In-reach, Independent, or a combination of both) would likely be dependent on the local health care context. Funding arrangements, assessment and eligibility to community services and the governance arrangements of the hospitals and community services would all be likely to influence which model(s) were utilised to meet the needs of older adults with ‘complex’ discharge needs.

In order to improve health care and patient and carer outcomes, the studies included in this review attempted to do this primarily through the augmentation of staffing arrangements. While staff play an integral role in the discharge of older adults from hospital into the community, given the complexity of problems associated with discharge across the interface, changes in staffing arrangements alone may be insufficient to significantly improve discharge outcomes. Common discharge problems across the interface, such as communication breakdowns [13,14,40,41], inadequate and delayed assessments [13,19] and delayed involvement of community services post-hospital discharge [19], extend beyond the control of individual staff members. These complex problems involve organisational and broader environmental issues, and as such, potential solutions are likely to require complex, multi-level and system-wide interventions.

Further targeted research is required into understanding community services' involvement in the discharge process of older adults from hospital into the community. This research needs to consider and report in detail on the following:

- Why the intervention was developed and its specific intent?
- How the specific discharge needs of the older adult, or group of older adults, were addressed by the intervention (e.g. those at risk of hospital readmissions).
- The key differences between the intervention and the usual care group.
- Viability of the researched intervention(s) within ‘normal’ resources (e.g. staffing, community availability, funding and governmental policies).
- How and when community services were engaged.
- The specific services delivered by the community services.
- How commonly reported problems in the literature like poor communication and communication breakdowns [13,14,40,41]; delayed and inadequate assessments of discharge needs [13,19]; poor organisation of community services [16,17] and delayed community services involvement after discharge from hospital [19] were addressed.
- The level(s) of the health care system which the intervention attempted to influence in order to improve the discharge process and the most appropriate research methods to use to research this.

Further research should also consider integrating a key set of outcome measures, which would enable pooling of data in future reviews. The key set of outcome measures should consider outcomes in relation to all stakeholders in the process (hospitals, community services and patient/carers) and the commonly reported consequences of problematic discharges between hospital and the community.

### Limitations of this review

There is currently a paucity of research into community service involvement in the discharge process. Thus, despite a comprehensive search, we were unable to draw firm conclusions regarding the effectiveness of the models of community services involvement in the discharge process.

The review was limited to English language studies. Restriction by language may result in bias, because there may well be effective models of care operating in non-English-speaking countries.

There were a number of conceptual difficulties in undertaking a systematic review into models of care, which has been highlighted in a previous review [42]. These included differing terminology around the discharge process, different health care systems, different professional titles (i.e. district nurse versus community nurse) and constructing a search strategy which was non-condition specific.

The literature also did not specifically describe the different components of care delivered or how these compared to the usual care group. Thus, it was not possible to identify which components of care were potentially linked with changes in outcomes, or whether potentially the same components of care were delivered in both the intervention and usual care group, just ‘badged’ under different names.

## Conclusion

This systematic review identified four models of community service involvement in the discharge of older adults from hospital. It is plausible that these models may be important in facilitating safe, effective and timely discharge from hospital. Further research is required which aims to better match older adults’ discharge needs with appropriate models of care, rather than assuming that older adults are a homogenous group which responds well to the one discharge model. Identifying appropriate and clinically practical models of community service involvement in discharge processes seems essential to ensure that the Australian health care system can better respond in a sustainable manner to the needs of a diverse and increasing cohort of older adults.

## Reviewers

**Jackie Cumming**, Professor, Health Policy and Management, School of Government, Victoria University, Victoria, New Zealand.

**David Perkins**, PhD, Professor of Rural Health Research, Centre for Rural and Remote Mental Health, Faculty of Health and Medicine, the University of Newcastle, NSW, Australia.

**Emily Piraino**, MSc, Psychogeriatric Resource Consultant – Algoma, North Bay Regional Health Centre, Ontario, Canada.

## Figures and Tables

**Table 1.**
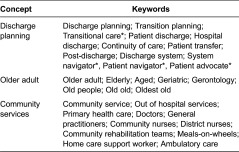
Full-search strategy used to identify studies for the review

**Table 2.**
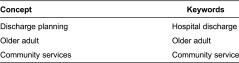
Abridged search strategy used to identify studies for this review

**Table 3.**
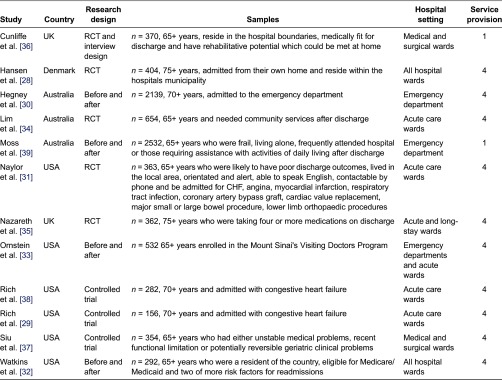
Characteristics of studies included in this review

RCT, randomised controlled trial; CHF, congestive heart failure.

Key: Community service provision

1. Those that provide interventions to maintain or optimise functional capacity and independence.

2. Services that provide slow stream rehabilitation.

3. Services that provide case management role situated in the community.

4. Those that provide planned, supported transfer of patients from hospital to community.

**Table 4.**
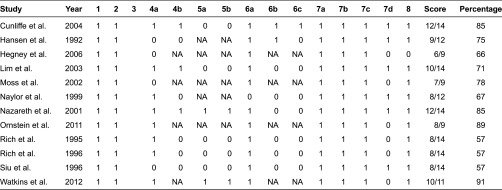
Quality scores of retrieved articles

Key to scoring the McMasters quantitative critical appraisal tool: All domains, except three, were scored. 1 for yes, 0 for no or not addressed, and the item was deducted from the overall score for not applicable.

1. Was the purpose stated clearly?

2. Was the relevant background literature reviewed?

3. Design (*not scored*)

4. Sample

4a. Was the sample described in detail?

4b. Was the sample size justified?

5. Outcomes

5a. Were the outcome measures reliable?

5b. Were the outcome measures valid?

6. Intervention

6a. Intervention was described in detail?

6b. Contamination was avoided?

6c. Cointervention was avoided?

7. Results

7a. Results were reported in terms of statistical significance?

7b. Were the analysis method(s) appropriate?

7c. Clinical importance was reported?

7d. Drop-outs were reported?

8. Conclusion was appropriate, given the study methods and results.

**Table 5.**
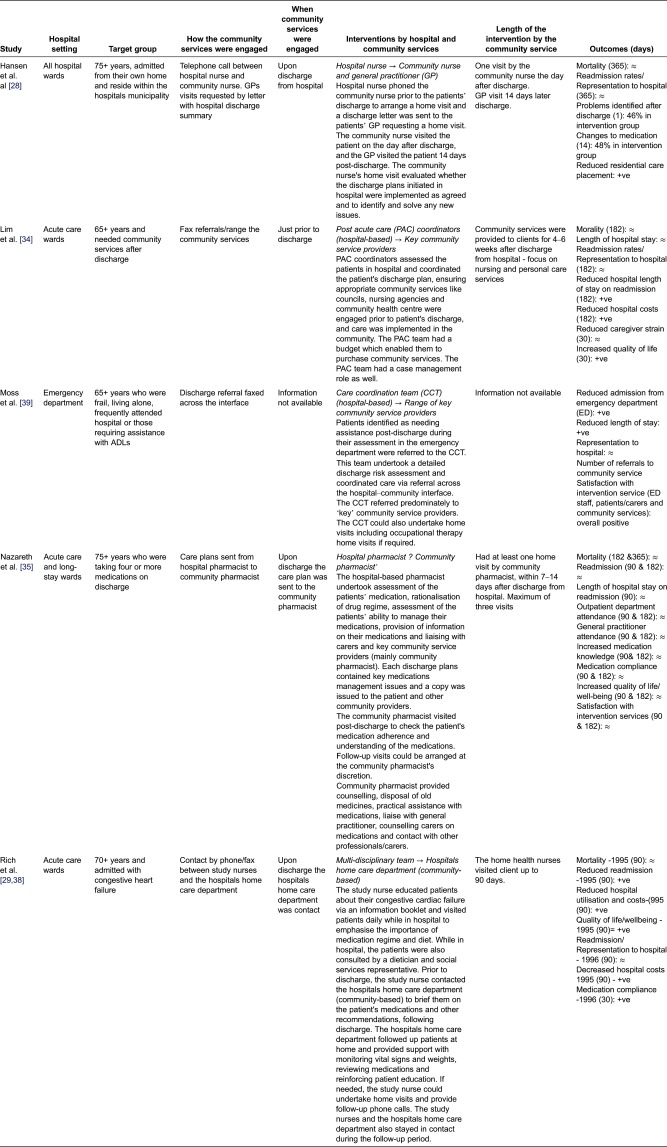
Summary of studies within the Virtual Interface Model

Key

Significantly positive +ve.

No significant difference≈.

Significantly negative −ve

Decreased but not analysed statistically ↓.

**Table 6.**
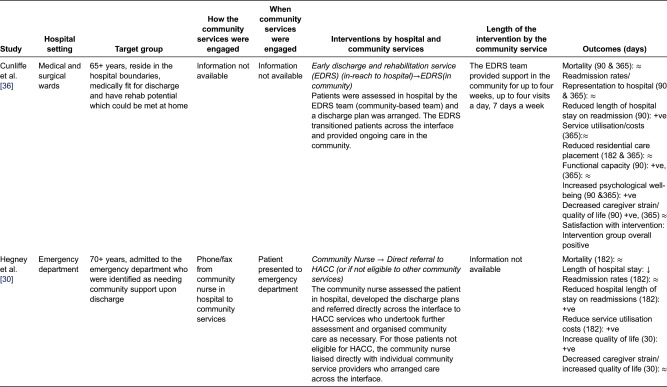
Summary of studies within the In-reach Interface Model

Key

Significantly positive +ve.

No significant difference≈.

Significantly negative −ve.

Decreased but not analysed statistically ↓.

**Table 7.**
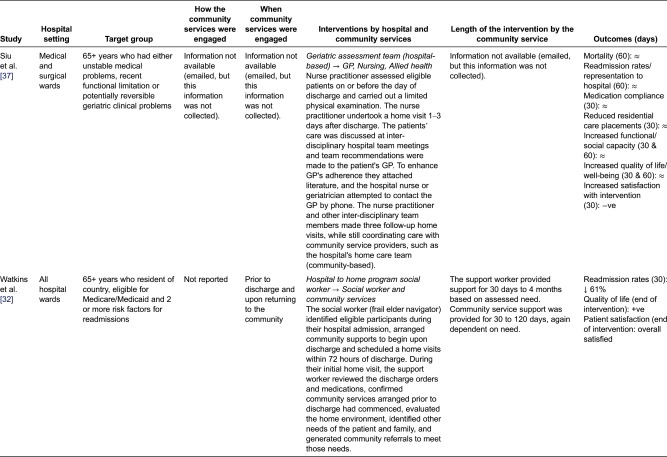
Summary of study in the Out-reach Interface Model

Key

Significantly positive +ve.

No significant difference≈.

Significantly negative −ve.

**Table 8.**
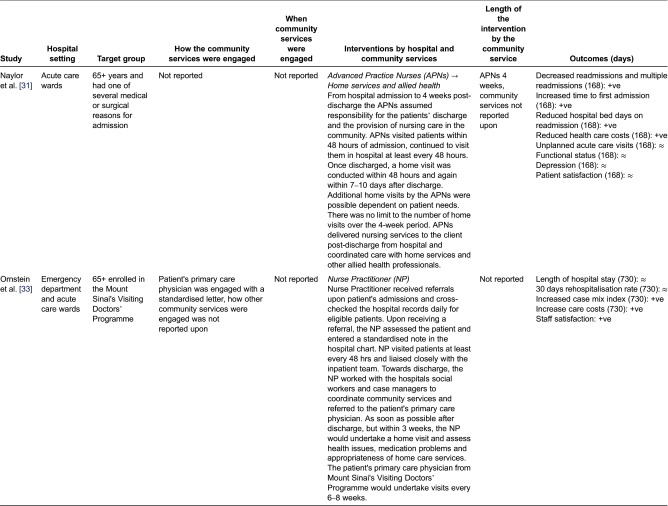
Summary of studies on Independent Interface Model

Key

Significantly positive +ve.

No significant difference≈.

Significantly negative −ve.
